# The Luxembourg Parkinson’s Study: A Comprehensive Approach for Stratification and Early Diagnosis

**DOI:** 10.3389/fnagi.2018.00326

**Published:** 2018-10-29

**Authors:** Geraldine Hipp, Michel Vaillant, Nico J. Diederich, Kirsten Roomp, Venkata P. Satagopam, Peter Banda, Estelle Sandt, Kathleen Mommaerts, Sabine K. Schmitz, Laura Longhino, Alexandra Schweicher, Anne-Marie Hanff, Béatrice Nicolai, Pierre Kolber, Dorothea Reiter, Lukas Pavelka, Sylvia Binck, Claire Pauly, Lars Geffers, Fay Betsou, Manon Gantenbein, Jochen Klucken, Thomas Gasser, Michele T. Hu, Rudi Balling, Rejko Krüger

**Affiliations:** ^1^Clinical and Experimental Neuroscience, Luxembourg Centre for Systems Biomedicine, University of Luxembourg, Esch-Belval, Luxembourg; ^2^Neurology, Centre Hospitalier de Luxembourg, Luxembourg, Luxembourg; ^3^Competence Centre in Methodology and Statistics, Luxembourg Institute of Health, Strassen, Luxembourg; ^4^Bioinformatics Core, Luxembourg Centre for Systems Biomedicine, University of Luxembourg, Esch-sur Alzette, Luxembourg; ^5^Integrated BioBank of Luxembourg, Dudelange, Luxembourg; ^6^Developmental and Cellular Biology, Luxembourg Centre for Systems Biomedicine, University of Luxembourg, Esch-sur Alzette, Luxembourg; ^7^Clinical and Epidemiological Investigation Center, Luxembourg Institute of Health, Strassen, Luxembourg; ^8^Department of Molecular Neurology, University Hospital Erlangen, Friedrich-Alexander University Erlangen-Nürnberg, Erlangen, Germany; ^9^Department of Neurodegeneration, Hertie-Institute for Clinical Brain Research and German Center for Neurodegenerative Diseases, Tübingen, Germany; ^10^Nuffield Department of Clinical Neurosciences, Medical Sciences Division, University of Oxford, Oxford, United Kingdom

**Keywords:** parkinsonism, cohort, longitudinal, stratification, deep phenotyping

## Abstract

While genetic advances have successfully defined part of the complexity in Parkinson’s disease (PD), the clinical characterization of phenotypes remains challenging. Therapeutic trials and cohort studies typically include patients with earlier disease stages and exclude comorbidities, thus ignoring a substantial part of the real-world PD population. To account for these limitations, we implemented the Luxembourg PD study as a comprehensive clinical, molecular and device-based approach including patients with typical PD and atypical parkinsonism, irrespective of their disease stage, age, comorbidities, or linguistic background. To provide a large, longitudinally followed, and deeply phenotyped set of patients and controls for clinical and fundamental research on PD, we implemented an open-source digital platform that can be harmonized with international PD cohort studies. Our interests also reflect Luxembourg-specific areas of PD research, including vision, gait, and cognition. This effort is flanked by comprehensive biosampling efforts assuring high quality and sustained availability of body liquids and tissue biopsies. We provide evidence for the feasibility of such a cohort program with deep phenotyping and high quality biosampling on parkinsonism in an environment with structural specificities and alert the international research community to our willingness to collaborate with other centers. The combination of advanced clinical phenotyping approaches including device-based assessment will create a comprehensive assessment of the disease and its variants, its interaction with comorbidities and its progression. We envision the Luxembourg Parkinson’s study as an important research platform for defining early diagnosis and progression markers that translate into stratified treatment approaches.

## Introduction

Even 200 years after the first description of the diagnosis of Parkinson’s disease (PD) (Dorsey et al., 2007), there are substantial gaps in our understanding of the underlying mechanisms and the complex clinical presentation of PD. The differential diagnosis can remain challenging, especially at the early stages of the disease; we still lack prognostic markers predicting the disease trajectory and the treatment remains symptomatic.

Consequently, strategies for defining novel treatment concepts and improving the diagnostic accuracy at the early stages need to account for the clinical and etiological heterogeneity of PD.

This clinical complexity defines the variable phenotypes of the disease, which are represented by a variable combination of different motor and non-motor symptoms and ranges from early onset forms with slow disease progression and only few axial symptoms to late-onset forms with early dementia and gait disturbance (Krüger et al., 2016). Non-motor symptoms receive more and more attention in the differentiation of subtypes of the disease as these can precede the diagnosis for years. Some of them have been therefore integrated in the research criteria for prodromal PD (Berg et al., 2015), and can be used to better stratify PD patients with implications on prognosis and treatment response (Sauerbier et al., 2016). Amongst these, cognitive impairment has gained more and more interest as the cumulative incidence of dementia in PD reaches up to 80% (Hely et al., 2008, cited by Yarnall et al., 2014). Here, it was shown that 42.5% of newly diagnosed PD patients present already with mild cognitive impairment (MCI), correlated with a with a decrease of Abeta42 and Abeta40 levels in CSF (ICICLE-PD study; Yarnall et al., 2014). As MCI increases the risk for dementia, more data on biomarkers for cognitive impairment is needed in order to enable accurate predictions for dementia. Rare atypical parkinsonian syndromes, like Progressive Supranuclear Palsy (PSP), Corticobasal Syndrome (CBS), or Multiple System Atrophy (MSA) (Levin et al., 2016) represent common challenges for differential diagnosis of PD, especially during early disease stages (Ali and Morris, 2015; Lehosit and Cloud, 2015). Follow-up is needed to definitely establish the diagnosis, and some patients may only convert after more than 10 years from PD to atypical parkinsonism (Petrovic et al., 2012). Currently, however, most cohort studies are excluding patients with undefined atypical parkinsonism (Mollenhauer et al., 2013; Szewczyk-Krolikowski et al., 2014), although cohorts including them may better describe the various possible disease trajectories.

Well in line with the etiological heterogeneity of the disease, an increasing number of genes and environmental risk factors have been identified, all playing a role in neurodegeneration in PD (van der Brug et al., 2015; Elbaz et al., 2016). However, these are still far from explaining the majority of PD cases, thus indicating the need of well characterized cohorts to better define the natural history of PD, to identify and validate biomarkers and to cluster subgroups of patients for clinical trials. This need in mind, a substantial number of observational studies in prodromal and clinical PD have been initiated during the last years (Lerche et al., 2015). Again, completeness of the clinical spectrum has not been achieved by the respective recruitment strategies, as most of these studies only included patients at the early disease stages (Lerche et al., 2015; Malek et al., 2015), while more advanced PD stages were underrepresented (Santos-García et al., 2016).

The risk for a recruitment bias is given, with inclusion of phenocopies (e.g., subjects without evidence for dopaminergic deficit, SWEDD) (Marshall et al., 2009), and persistent lack of information on the natural disease progression in more advanced stages of PD. Thus, inclusion of *all* disease stages and longitudinal follow-up studies are crucial to address these knowledge gaps.

Moreover, the correlation of available genetic data with the spectrum of clinical symptoms of PD is still limited (Grünewald, 2013). Existing studies focus either on genotyping with limited availability of clinical data [e.g., age, gender and year of disease onset in GWAS studies (Simón-Sánchez and Gasser, 2015)], or on clinical phenotyping, with comprehensive clinical data, but limited genetic information [i.e., DeNoPa (Mollenhauer et al., 2013)]. In order to bridge this gap, studies combining deep clinical phenotyping and a comprehensive assessment of genetic and biological data are needed. Finally, in order to achieve significant sample sizes that allow for validation across cohorts, a harmonization in terms of scales, and/or, study design is required. Already at the planning stage of a study, data harmonization with other recruiting centers should be envisioned to validate findings from different studies in a larger “collective” of patients.

Given the fact that the diagnostic and progression evaluation of PD is still left to be fundamentally based on the clinical assessment as defined by International Parkinson and Movement Disorders Society (Postuma et al., 2015a), the urgent need for biomarkers supporting the diagnosis, progression evaluation, response to the therapy and finally specific subtype distribution has become apparent. Biomarkers could be generally summarized into 6 groups: *diagnostic* including the prodromal diagnostic biomarkers as well *early stage disease biomarkers, progression biomarkers* along with *staging biomarkers, theragnostic biomarkers* reflecting the response to treatment and finally *stratification* biomarkers as a base for translational research and precision medicine with the ultimate goal to implement the disease-modifying treatments.

Still ongoing research in biomarkers provides discrepancies between the stratification of PD-subtypes based on clinical phenotypes rather than biomarker-driven stratification. For example, in the search for a diagnostic biomarker in CSF using the data based on PPMI and DeNoPa cohort (Kang et al., 2016; Mollenhauer et al., 2016), the CSF biomarkers of clinically defined phenotypes have provided conflicting results with substantial overlap with control group (Espay et al., 2017). Moreover, the stability of the clinically defined phenotypes seems to vary over time (Simuni et al., 2016) and therefore suggest a low accuracy in defining reliable biomarkers. As for the prognostic biomarkers, the presence of sleep disorders such as REM sleep Behavior Disorder (RBD) has been widely investigated and has been found to be associated with severe hyposmia, higher frequency of non-motor symptoms, particularly depressive syndromes and generally poorer prognosis (Zhou et al., 2016). To address the above mentioned limitations, we designed the Luxembourg Parkinson’s study, focusing on the recruitment of patients with PD and atypical parkinsonism at all disease stages and directly planning for a long longitudinal a follow-up under real world conditions.

Our study represents an ideal exploratory, *a priori* unbiased by design cohort using a comprehensive longitudinal clinical assessment accompanied by omics-based molecular fingerprints analysis and combined with genotyping that will eventually allow for a biomarker-driven stratification of PD in a well-defined population.

Such a multidimensional approach ranging from genes and complex molecular fingerprints to the longitudinal clinical assessment promises to facilitate the detection of PD subtypes and the disease-specific biomarkers on the way to the precision medicine model. As a consequence, the well-defined subtypes of PD are key to success in future clinical trials implementing the disease modifying drugs.

This description of the program outlines the major axes of data, strategies, and research approaches, in the context of a national health initiative but also as an international source for sharing and collaborative efforts in neurodegeneration research.

## Materials and Methods

### Design

#### Type of the Study

The Luxembourg Parkinson’s study is a nation-wide, monocentric, descriptive, observational, longitudinal-prospective study with an annual follow-up of patients. Control subjects will be followed up after 4 years. The baseline evaluation is designed as case-control study, with an initial recruitment period over 4 years.

#### Specific Goals

We focus on the comprehensive population-based recruitment of all patients with parkinsonism in Luxembourg and the surrounding ‘Greater Region’ (including the German, French, and Belgian border regions). Recruitment and communication strategies are tailored to the multilingual background of participants, and include Luxembourgish, German, French, English, and Portuguese as most popular languages.

Our specific tools allow us to focus on five main objectives (Table 1).

**Table 1:** Objectives.

Objective	Tools	Endpoints
Clinico-genetic stratification of parkinsonism	−Deep clinical phenotyping (motor and non-motor) with annual follow-up −Genotyping (NeuroChip; Blauwendraat et al., 2017) −Investigation of exposure factors −Different disease stages	Disease history under real-world conditions Stratification markers Progression markers Novel disease markers

Differential diagnosis of atypical parkinsonism	−Inclusion of all atypical PD forms −Inclusion of patients with conversion of diagnosis in follow-up of	Conversion markers Differential markers (IPD/atypical PD) Early markers for the development of atypical parkinsonism

Identification of differential cognitive profiles in PD and atypical PD	−Extensive cognitive phenotyping (five cognitive domains) −Investigation of MCI/PDD MDS Level 2 criteria −Tests to investigate cognition in atypical PD forms −Assessment in different languages in a multilingual population −Establishment of population specific normative data	Cognitive markers for PDD and DLB MCI-subtypes MCI-subtypes (executive, visuo-spatial, amnestic dominant vs. multiple domain (Kalbe et al., 2016) Early diagnostic markers for atypical PD forms Risk/protective factors for dementia

Dissection of association between gait disturbances and cognition	−Detailed assessment of gait including sensor based measures [mPower (Trister et al., 2016), instrumental gait analysis (Klucken et al., 2013; Schlachetzki et al., 2017)] −In-depth and a continuous information collection about disease progression −Various aspects of gait can be linked to various cognition factors	Validation of sensor based assessment for future clinical trials Cognitive predictors for gait impairment

Definition of vision as an early disease marker	−Detailed assessment of vision including color discrimination, contrast sensitivity, and facial emotion recognition (Diederich et al., 1998, 2010; Pieri et al., 2000; Hipp et al., 2014)	Vision as an early marker of PD Facial emotion recognition as a marker for PD

#### Harmonization Strategy

Emphasis was put on harmonization of the datasets with ongoing international cohort studies for comparability and cross validation, thereby increasing statistical power of the planned analyses. Therefore, the diagnostic criteria and scales applied in our study, have been aligned with already existing international cohort programs, e.g., DeNoPa (Mollenhauer et al., 2013), Oxford PD Centre (OPDC) (Lawton et al., 2015); PPMI (Marek et al., 2011), GEoPD (Puschmann et al., 2015), as described previously (Lerche et al., 2015). Harmonization rates are shown in Table 2.

**Table 2:** Studies and percentage of common assessments with HELP-PD.

Study name	% of tests integrated in HELP-PD
LONG-PD (GEoPD, https://www.geopd.net)	93%
OPDC (http://opdc.medsci.ox.ac.uk)	73%
PPMI (https://www.michaeljfox.org)	67%
DeNoPa (visit 1) (http://www.denopa.de/)	56%

To further increase the inter-comparability across the above mentioned studies, we implemented different tests with validated conversion procedures, i.e., MoCA/MMSE (van Steenoven et al., 2014), “Sniffin’ Sticks”/University of Pennsylvania Smell Identification Test (UPSIT) (Malek et al., 2017), UPDRS/MDS-UPDRS (Goetz et al., 2012).

#### Communication Strategies

The implemented communication strategy aims to raise awareness and spread information about the cohort study to medical professionals, patients and the general public in Luxembourg and the Greater Region. All communication materials have been made available in five languages (Luxembourgish, German, French, English, and Portuguese) with German and French being by far the most favored languages. To reach the communication objectives, different communication channels have been established (Table 3).

**Table 3:** Communication channels developed.

Web	Information
Dual-entry website (www.parkinson.lu)	Research and medical professionals Patients and control subject
Facebook site (Parkinson: Recherche au Luxembourg)	Study update
Use of established social media channels from partner institutions	
Comprehensive NCER-PD video	In German and French explains the aims of the program and gives an overview of the participation steps and subsequent research projects

**Paper**	

Multilingual flyer and posters	Information about PD, information about study participation
Fact sheets	Information on disease-related subjects
Bi-annual print newsletter	Regular update of study and Parkinson’s disease care and research in general

### Recruitment Strategy

All the subjects have signed a written informed consent, and the collection has been approved by the National Ethics Board (CNER Ref: 201407/13) and Data Protection Committee (CNPD Ref: 446/2017).

A clinical steering committee composed of different health professionals from Luxembourg involved in PD care supervise the recruitment procedures.

#### Sample Size Calculation

Based on our sample size estimations, we will include 800 patients with idiopathic PD or atypical parkinsonism, as well as 800 healthy control subjects.

The estimated prevalence and annual incidence of PD in Luxembourg are 565–1,356 and 57–100, respectively, based on available epidemiological data from other European countries (von Campenhausen et al., 2005). Atypical forms of PD are expected to be rare. For instance, for PSP we can only expect 7–25 patients in Luxembourg based on available data on prevalence (von Campenhausen et al., 2005).

Assuming a type I error rate of 5% two sided and a power of 80%, 800 patients in each group would allow finding a significant difference between groups. For instance, a two group χ^2^ test with a 0.050 two-sided significance level will have 80% power to detect the difference between a smaller proportion, π_1_, of 0.010 and a larger proportion, π_2_, of 0.031 when the sample size in each group is 800.

For instance if the proportion of MoCA < 26 is as low as 0.031 in the PD group and 0.010 in the non-PD (control) group, 800 patients in each group would allow to show a statistically significant difference in cognitive impairment in the PD group.

Proportions of this estimated factor ranging from 1 to 20% in the control group and the corresponding proportions in the PD group for which a true difference would be detectable with the target power are presented in Table 4.

**Table 4:** Sample size for baseline comparisons.

Smaller proportion, πa	0.01	0.05	0.1	0.15	0.2
Larger proportion, πb	0.031	0.087	0.147	0.205	0.26

However, for the within-cohort comparisons equality in numbers of any subgroups being compared cannot be assumed. Therefore, a minimum of 100 has been set for the smaller subgroup of two being compared (with 700 for the larger).

Simulations of a series of differences between groups for a particular character of interest gives a power of 82% that the corresponding differences that can be detected. The Table 5 illustrates the situation where the larger probability is in the larger group and the situation where the larger probability is in the smaller group with the corresponding differences that can be detected.

**Table 5:** Sample size for longitudinal within PD cohort comparisons.

	Larger Prob. In larger group			Larger Prob. In smaller group		
Prob. group 1	Prob. group 2 (*N* = 700)	Difference	Attained power	Prob. group 2 (*N* = 100)	Difference	Attained power
0,05	0,134	0,084	81,9	0,145	0,095	81,8
0,1	0,206	0,106	81,9	0,206	0,106	81,9
0,15	0,271	0,121	81,9	0,271	0,121	81,9
0,2	0,332	0,132	81,8	0,332	0,132	81,8
0,25	0,391	0,141	81,8	0,391	0,141	81,8

Therefore if we assume that the PD group is divided in two categories (Hoehn and Yahr I and II or III and IV) with probability of progression of 0.05 observed in the smaller category (*n* = 100) and probability of progression is 0.134 in the larger category, the difference of 0.084 could be shown with a power of 81.9%. Genetic data would also be used here, whereby the group of carriers of a specific variant with an observed frequency of progression compared to the non-carriers group.

#### Inclusion and Exclusion Criteria

##### Patient group

To be classified as idiopathic PD, patients must meet the inclusion criteria proposed by the United Kingdom Parkinson’s Disease Society Brain Bank Clinical Diagnostic Criteria (Hughes et al., 1992).

Patients who do not fulfill the proposed criteria will be classified as unspecified PD or as atypical PD based on the respective criteria. In the atypical PD subgroup, further classification will include subtypes, including PSP (Litvan et al., 1996; Höglinger et al., 2017), MSA (Gilman et al., 2008), CBS (Boeve et al., 2003) or vascular parkinsonism (VP) (Zijlmans et al., 2004), based on internationally established criteria. All diagnostic classifications will be regularly updated. Patients with essential tremor are excluded from the patients group, and included into the control group. They may convert into typical PD and would then qualify for the inclusion into the patient group (Unal Gulsuner et al., 2014; Laroia and Louis, 2011).

Patients with a secondary cause of parkinsonism (e.g., normal pressure hydrocephalus, toxic parkinsonism, medication-induced parkinsonism, symptomatic parkinsonism due to structural lesions) are excluded. Here, separation was based on established diagnostic criteria that include clinical symptoms as well as available clinical imaging results. Whereas normal pressure hydrocephalus may still be clinically over suspected (Espay et al., 2017), and presents with parkinsonism, gait disturbance, urinary symptoms, as observed for VP, the cardiovascular risk profile and the typical imaging findings with vascular lesions vs. symmetric enlargement of ventricles and diapedesis of CSF defines the difference of both secondary causes of parkinsonism (Rektor et al., 2018).

##### Healthy control group

Healthy control subjects are recruited and matched for age and gender via continuous statistical calculations. Subjects with a neurodegenerative disease are excluded (c.f. Table 6). Controls include spouses of patients and unrelated volunteers who are partially recruited from a pool of healthy controls previously participating in independent Luxembourgish observational studies such as the ORISCAV-LUX study (Crichton and Alkerwi, 2014) or EHES-LUX, the 2013 Luxembourgish part of the European Health Examination Survey (EHES) study (Kuulasmaa et al., 2012; Ruiz-Castell et al., 2016). The Frequency of PD patients stratified by age, gender, residence and BMI is calculated at regular intervals and the recruitment of healthy control subjects is subsequently oriented toward having a match between newly recruited PD patients and healthy controls and can be adjusted via available subjects in the previous described epidemiological studies.

**Table 6:** Inclusion and exclusion criteria.

	Patients	Controls
Inclusion criteria	Diagnosis of parkinsonism	Subjects without ND disease
	>18 years of age	>18 years of age
Exclusion criteria	Absence of parkinsonism	Presence of neurodegenerative disorder
	Symptomatic parkinsonism	
	Active cancer	Active cancer
	Pregnant women	Pregnant women
	Limited capacity of consent on the part of the subject, if there is no legally determined tutor	

#### The Flexible Participation Concept

**FIGURE 1:**
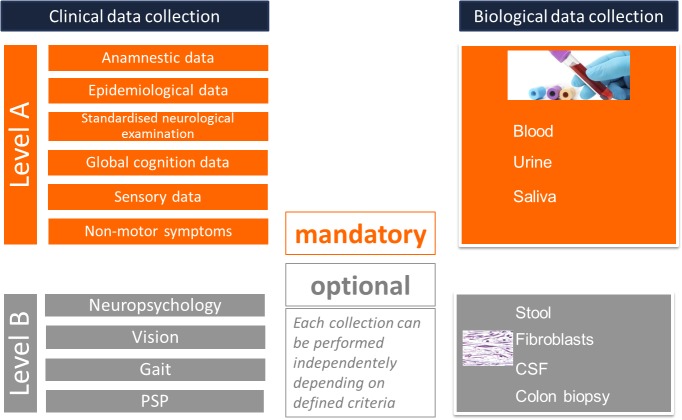
Flexible participation concept.

To account for potential variable motivation of the heterogeneous population in Luxembourg and to improve adherence to our study, we implemented a *flexible participation* concept. Here, the participants can choose between different participation levels concerning clinical assessments and biosampling, by offering a basic assessment level (Level A) that is mandatory for all participants, and an optional assessment level (Level B) including a variety of focused assessments detailed below and focusing more specifically on vision, gait or specific aspects of PSP. Level B also proposes more invasive biosampling such as lumbar puncture or skin biopsy (Figure 1). Level A assessment and biosampling are carried out during one visit of 2–3 h. The Level B tests and the optional biosampling are performed during independent visits not exceeding 2 h in total (Figure 1).

#### Mobile Recruitment Team

Participants are recruited either at the research center site or at different sites in Luxembourg and the Greater Region defined as “hubs” that are attended by a mobile recruitment team (“flying team”) allowing participants to be recruited closer to their home area avoiding transport issues.

In case a patient cannot join for a follow-up visit, neither at the center, nor by the mobile recruitment team, e.g., because of extremely reduced mobility, or living too far away from the recruitment area, a standardized telephone or Skype questionnaire is proposed containing a reduced assessment of Level A.

### Assessments

#### Clinical Phenotyping

*Motor function* and motor complications are assessed by using self-reported questionnaires, rating scales and standardized objective test measures. Sensor based assessments have been integrated to address multiple variables of bradykinesia and gait (Table 7). We assess *non-motor*, e.g., *dysautonomic symptoms* and their impact on activities of daily living and quality of life by using self-reported questionnaires (Table 8). *Global cognitive function* is assessed with the MoCA test. Additionally each of the five cognitive domains (executive, memory, attention, visuo-spatial, and language), is assessed by two tests according to the Level 2 criteria for sub-typing classification for MCI (Litvan et al., 2012) and PD with dementia (PDD) (Dubois et al., 2007). The executive functions with the sub-domains planning, initiation, inhibition, set shifting, and conceptualization are explored in detail. In the visuo-spatial domain our test battery will allow to differentiate between perceptive and constructive abilities (Table 9). Finally the assessment of *sensory function* encompasses tests for *odor and vision* (Table 10).

**Table 7:** Assessment tools for motor function.

	Self-reported	Rating scale	Objective measure
Global motor		^+^MDS-UPDRS III and ^+^IV	
Bradykinesia		^+^MDS-UPDRS III	mPower tapping activity (Trister et al., 2016)
Fine/gross motor and coordination			^+^Purdue Pegboard^∗^ (Tiffin and Asher, 1948)
Gait-general		^+^MDS-UPDRS III	Timed up and go mPower walking activity
Freezing of gait	Freezing of Gait Questionnaire New freezing of Gait Questionnaire	^+^MDS-UPDRS III Freezing of gait assessment course (Ziegler et al., 2010)	Instrumental gait assessment (Klucken et al., 2013)
PSP specific motor dysfunction		Unified PSP Rating Scale (Golbe and Ohman-Strickland, 2007)	

*^+^Included in Level A (mandatory) assessment*.

**Table 8:** Questionnaires for non-motor symptoms in PD.

	Function/symptom	Test
Global	General non-motor symptoms	^+^Parkinson’s Disease Non-Motor Questionnaire (PD-NMS)
Psychiatric symptoms	Depression	^+^Beck Depression Inventory (BDI)
	Apathy	^+^Starkstein Apathy Scale (SAS)
Dysautonomic function	Including, e.g., constipation, daytime somnolence, symptomatic hypotension, erectile and urinary dysfunction	^+^SCOPA-AUT
Sleep	General	^+^Parkinson’s Disease Sleeping Scale (PDSS)
	REM sleep Behavior Disorder (RBD)	^+^RBD Screening Questionnaire (RBDSQ)
Quality of life	Eight disease-related issues: mobility, activities of daily living, emotional well-being, stigma, social support cognitions, communication, and bodily discomfort	^+^Parkinson’s Disease Quality of Life Questionnaire (PD-QoL-39)

*^+^Included in Level A (mandatory) assessment*.

**Table 9:** Tests used to assess cognition.

Domain	Function	Test
Global cognition		^+^Montréal Cognitive Assessment (MoCA)
Attention/working memory	Visual attention	^+^TMT^∗^-A
	Divided (visual/auditory attention)	TAP^∗∗^-Divided Attention
	Visuospatial short-term memory	Block spans (forward)
	Auditory short-term memory	Digit spans (forward)
	Visuo-spatial working memory	Block spans (backward)
	Auditory working memory	Digit spans (backward)
Executive	Mental flexibility/set shifting	^+^TMT-B TAP-Flexibility Set Test (Isaak)
	Automatic response inhibition	STROOP test (Kaplan) TAP-Go/No-go
	Planning	^+^Clock Test – Placement of numbers
	Conceptualization	Similarities (^+^MoCA, FAB)
	Frontal functions	Frontal Assessment Battery (FAB)
	Response initiation	^+^Letter Fluency F (MoCA) Letter Fluency S (FAB)
Memory	Verbal learning + episodic memory consolidation	Immediate and delayed word list recall (CERAD)
		Word Recognition (CERAD)
Visuospatial	Visuo-perception	Benton’s Judgment of Line Orientation
	Visuo-construction	^+^Wire Cube (MoCA)
		Interlocking Pentagons (MMSE)
		^+^Clock Test (MoCA)
Language	Language access	Category Fluency (animals)
		GREMOTs denomination of 36 substantives and 36 verbs

*^+^Included in Level A (mandatory) assessment. ^∗^Trail Making Test, ^∗∗^Test of Attention Performance*.

**Table 10:** Tests used to assess sensory function.

Function	Test
Odor identification	^+^Burghart Sniffin’ Sticks – 16 items identification test
Color discrimination	Farnsworth Munsell 100 Hue test
Contrast sensitivity	Pelli Robson Letter Chart
	Kybervision Contrast Sensitivity (Beaudot, 2009)
Facial emotion recognition	Ekman 60 Faces Test

*^+^Included in Level A (mandatory) assessment*.

If available from the clinical records, the information on previous clinical imaging (CT, MRI, DaTSCAN^TM^) was recorded in the electronic case report form (eCRF).

#### Sensor Based Measures

Device-based assessments (DBA) allow the objective longitudinal registration of relevant short term and gradual changes related to disease stage and progression of a patient’s clinical state. These changes may sometimes remain undetected in a conventional, “snap-shot” clinical setting.

These technologies provide an objective, time- and cost-effective approach and initial data from stand-alone mPower in the United States is promising (Figures 1, 3 d+e), however, the validation and correlation of sensor-based data with standardized clinical assessments in large, well-described cohorts remain a major need to translate into clinical decision support. We focused on two strategies, (i) a mobile phone application capturing data from the home environment of participants and (ii) a gait sensor used for lab-monitoring under controlled conditions at the recruitment hub.

**FIGURE 2:**
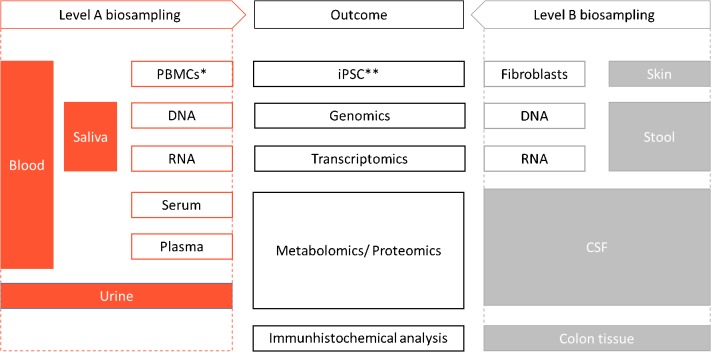
Omics assessment. ^∗^PBMC: Peripheral Blood Mononuclear Cell, ^∗∗^iPSC: induced pluripotent stem cells.

**FIGURE 3:**
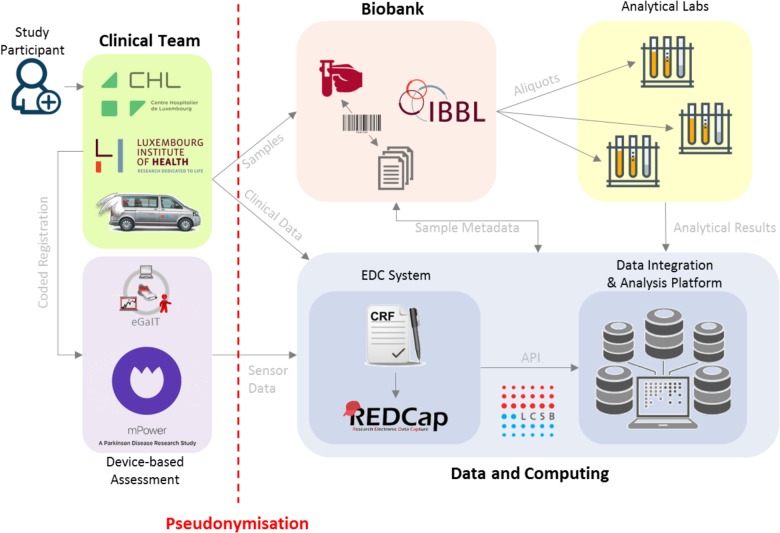
Data and sample flow in ND-collection. Subject personal data is securely collected and only the clinical team has access to that data. The clinical team generates a primary pseudonym and binds it to the subject’s personal record. The clinical data is deposited into REDCap, the Electronic Data Capturing (EDC) system, along with the pseudonym and the barcodes of the samples which are also recorded in the REDCap system. Sample Annotations go into the Data Integration and Analysis Platform which is a part of the Data and Computing Platform hosted at the LCSB in a secure data center. Within the Data and Computing Platform at the LCSB, the pseudonymized clinical data from REDCap is accessed by the Data Integration and Analysis Platform via an Application Programming Interface. As part of the direct clinical assessment, the kinetic gait data from the shoe sensors is deposited using the primary pseudonym generated by clinical team at the PRC.

For the first, we integrated the mPower application into the database of our Luxembourg Parkinson’s Study to add to the deep phenotyping of our cohort. This application combines a traditional survey-based approach with data gained through continuous sensor-based measurements, as well as task-based assessments (Bot et al., 2016). Anonymized longitudinal data from the application will be then correlated with longitudinal clinical and biological data in our database. It is an objective, time- and cost-effective approach, which will allow (i) to offer a more direct participation in our research, (ii) to define participant’s adherence to new technologies and (iii) to validate sensor-based algorithms with clinical data from standardized assessments in large cohorts of patients.

Wearable sensors were integrated by using a shoe equipped with a sensor to assess different aspects of gait during standardized gait tasks [instrumental gait analysis (Klucken et al., 2013; Schlachetzki et al., 2017)]. This lab-monitoring approach is implemented in the clinical visit and therefore the sensor-based assessment is paired with a structured clinical assessment of gait (Table 7), in order to allow for validation. Therefore a technological environment for capturing data and transferring to the study database was developed. In a second step these sensor-based gait monitorings should be transferred into the home environment of patients, to provide more ‘real life’ kinematic data allowing for classification of patients.

The implemented technologies will be a first step toward rater-independent appraisal of parkinsonian symptoms and support stratification of patients into subgroups.

#### Questionnaires on Environmental Factors

*Environmental exposure* data are collected through a modified version of the self-administered questionnaire with reference to the PD Risk Factor Questionnaire (PD-RFQ-U) Epi Info^TM^ | CDC^1^ of which the main questions have been extracted. We thus assess the exposures potentially relevant in our cohort such as caffeine, tobacco, alcohol, pesticide, anti-inflammatory agents, and hormonal medications. Residential and occupational history, physical activity and body habitus are evaluated by the same questionnaire. *The Family history* addresses PD, essential tremor, Alzheimer’s disease (AD) and more generally, dementias.

Broad genetic analyses for PD-associated mutations will be performed using NeuroChip technology (Blauwendraat et al., 2017), a genotyping array that allows to analyze 306,670 variants and it provides a manually curated custom content comprising 179,467 variants. This approach allows to screen for all currently known genetic risk variants for different neurodegenerative diseases, including PD, Dementia with Levy Bodies (DLB), AD, and Amyotrophic Lateral Sclerosis (ALS).

#### Assessment of Omics

Biospecimens are collected from patients and healthy controls at each visit using standardized collection kits, with blood, urine, saliva as part of the mandatory sampling (Figure 1). In addition, optional specimens including stool, skin biopsies and CSF can be collected. From the different biomaterials, we are able to derive iPSCs and different omics (Figure 2). Biospecimens are processed following Standard Operating Procedures (SOPs) (for details, see Supplementary Tables A, B) (Lehmann et al., 2012). All samples are stored at the Integrated BioBank Luxembourg (IBBL) and the details of storage conditions as well as all sample related annotations are captured in electronic databases. As sample quality is critical for the reproducibility and reliability of experimental results, IBBL has implemented validation and quality checks at all critical steps. They are continuously updated and extended, as needed (see Supplementary Tables).

### Data Analysis and Management

#### Endpoints

The data-driven and not hypothesis-driven analysis should allow us to detect yet unknown biomarkers identifying clusters or subgroups of patients with specific clinical trajectories, possibly coupled to defined omics’ characteristics. The longitudinal design of our study should allow further validation of the identified biomarkers. Specifically, the presence of certain markers at baseline will be correlated with the progression of the disease and to its different clinical patterns.

Besides standard statistical approaches (distributions, correlations, or independence tests), we are planning to employ well-grounded machine learning methods integrated into our data exploration and analytic platform, Ada^2^ using Spark ML library. This covers a wide variety of classification, regression, clusterization, feature selection, normalization, and time-series processing routines. We opted for Spark since it is a popular computational grid library for an efficient large-scale data processing and analysis. Ada’s computational infrastructure together with a convenient UI opens the advanced analytics and machine learning to a diverse group of researchers, clinicians, and statisticians.

#### Data Management

To provide sustained resource for research on PD, we implemented an advanced IT infrastructure that accounts for the heterogeneity of data (e.g., clinical, epidemiological, and biological) and the amount of data requiring an adapted strategy for big data management and visualization.

*All* clinical data and biosample metadata are collected and managed using electronic data capture tools developed and maintained by the LCSB (Harris et al., 2009) (Figure 3).

REDCap is a web-based application designed to support data capture for research studies. We have developed an eCRF in the REDCap framework that allows for centralized storage, high security and cost savings when compared to traditional paper-based approaches. In order to make it secure, our PD REDCap instance is encrypted, site restricted and controlled access with two-factor authentication.

A reporting system (Ada), developed in-house, provides key infrastructure for secured integration, visualization, and analysis of heterogeneous clinical and experimental data through the study. The platform currently manages anonymized data sets associated with clinical research pulled from REDCap system, biosampling-related information provided by IBBL, and kinetic data from mPower mobile application and gait sensors. As Ada also hosts DeNoPa study clinical data (three visits, Mollenhauer et al., 2013), it is a unique tool for future cross-study analyses and validations. DeNoPa dataset was therefore translated from German to English, curated for content and harmonized with the our eCRF.

Ada’s main features include a convenient web interface for dataset exploration and filtering, and configurable views with tables and charts showing basic statistics, such as, distributions, scatters, correlations, and box plots. To define dataset’s metadata Ada provides an editable dictionary, and a category tree with drag-and-drop manipulation [i2b2 – Informatics for Integrating Biology and the Bedside (Murphy et al., 2009, 2010)]. Furthermore, Ada facilitates robust access control through state-of-the-art authentication layer, and user management with fine-grained permissions.

The curated datasets are also integrated into a dedicated tranSMART system that supports cohort based integrated analysis and hypothesis generation.

#### Quality Management

Clinical assessments via raters experienced in the diagnosis of movement disorders still imply the risk of interrater variability. Here apparently the early stages of PD with only mild clinical symptoms impose the highest challenge for uniform rating results (Goetz et al., 2004). In order to assure high data quality and minimize interrater variability we integrated a constant benchmarking against the MDS-UPDRS training videos and regularly perform joint ratings by two independent staff members. Furthermore, we regularly perform internal video-assisted training sessions for the use of the assessment tools addressing difficult cases to ensure adherence to standardized procedures by all raters from the team.

Additionally, the REDCap system includes constraints in data fields and alerts in case of uncompleted data fields. Moreover, an independent study monitor performs regular source data verifications as well as verifications of completeness of predefined essential data.

Our REDCap database system is set up for sharing and harmonizing clinical and experimental data across different international sites. Moreover, a subset is available as minimal dataset within the Genetic Epidemiology of Parkinson’s disease (GEoPD Consortium^3^) providing data ownership for individual sites, but also options for joint analyses along harmonized datasets.

## Results

So far, we have included 498 patients and 520 healthy control subjects according to the recruitment plan. Ninety-four (14.1%) patients have been assessed by the flying team at one of our recruitment hubs. Currently, the recruitment numbers correspond to 101.8% of the initial recruitment plan and indicates the efficiency of our strategy.

At yearly follow-up, 229 patients have accomplished a second, and 92 a third visit. Over all the visits, 38 patients (8.9%) have been lost to follow-up for a given visit. 94 (14.1%) patients have been assessed by the flying team at one of our recruitment hubs. The reduced telephone questionnaire has been performed in 11 patients for the first, and in 10 for the second follow-up. Within the interval of 29 months a total of 13 patients deceased, nine after their first and four after their second visit. The reasons for death were pneumonia (one case), cardio-respiratory failure (three cases), septicemia (one case), or not available (seven cases).

The participation in Level B assessments and optional sample collection are relatively high even if proposed optionally (e.g., 896 stool samples) (Table 11).

**Table 11:** Participation numbers in Level B.

	PD patients *N*			Healthy controls *N*	Total *N*
	Visit 1	Visit 2	Visit 3		
Level B neuropsychology	192	29	4	252	477
Level B gait	137	99	63	87	386
Level B vision	75	37	7	95	214
Level B PSP assessment	8	0	0	0	8
Stool	280	162	60	394	896
Skin biopsy	58	26	11	69	164

At this stage, the patient group is composed of 422 (84.6%) IPD (44 with PDD), 7 (1.4%) DLB, 4 (0.8%) MSA, 6 (1.2%) CBS and 26 (5.2%) PSP patients. Our inclusion criteria allowed thus to include already 15% patients with confirmed or probable atypical forms of parkinsonism, and at follow-up, the first converters form IPD to PSP could already be identified.

We achieved a representation of patients form all disease stages, including the advanced ones. More than a third of the patients have H&Y > 2 (37.2%) and still 12.6% show H&Y > 3. Median H&Y stage is 2 with a range from 1 to 5 (46.7% with 2). Mean disease duration is 6.45 ± 5.44 years with a range from *de novo* patients to 30 years of disease duration.

Concerning the socio-cultural level, the accomplished education-years in the patient group reach from 1 to 30 years (mean: 12.57 ± 4.1).

Except for the Luxembourgish language, which is the most represented first language in our population with 62%, German and French being the second most represented (13% and 15%, respectively). In most of the population (82%) they are either the first or the second best spoken language. This means that we cover 82% of our population with the use of assessment tools in German and French. The remaining 18% proportion can be covered by the use of English assessment tools (English as first or second language, 16%). Only a minor proportion (2%) cannot be reached by any of these three languages. Here, we adapt by orally translating parts of the assessment in their language.

In terms of harmonization of data we successfully aligned data dictionaries within the REDCap databases with the Oxford Parkinson’s Disease Centre (OPDC; United Kingdom) and the Tübingen Parkinson’s Programme (ABC-PD; Germany). These are currently used for cross-validation of neuropsychological features and questionnaires across different study sites.

## Discussion

The Luxembourg Parkinson’s Study aims to combine comprehensive and longitudinally collected clinical data with emerging experimental data and biomarker programs. The aim is to bridge the gap between molecular information and clinical phenotype in PD, by integrating multidisciplinary competences in the area of clinical research, biomedical IT, computational modeling, and fundamental research including innovative technologies.

Our study in Luxembourg and the Greater Region exemplifies the feasibility of a cohort program with both deep clinical phenotyping and high quality biosampling on parkinsonism in an environment with limited exposure to clinical research. The success of the adapted recruitment strategy, including the concept of flexible participation, is reflected by the achievement of the initial recruitment goals, the high level of adherence of the participants and even the high level of participation to Level B. Here, the concept of a network structure represented by a ‘center without walls,’ involving stakeholders from different areas of healthcare (hospitals, private practices, nursing homes, different health professionals, and representatives from various research institutes, was largely accepted and contributed to the success, instead of a geographically limited ‘center with walls.’) The pioneering character within the implementation process also relates to the administrative framework and includes an Institutional Review Board (IRB) procedure that contributed to the development of a first IRB guideline for the handling of genetic incidental findings occurring during next generation sequencing in Luxembourg. Another added value was the establishment of data protection procedures including the possibility of exchange of pseudonymized data within scientific collaborations. Our study adds to current cohort designs that either perform deep genetic stratification in large cohorts of PD patients with only limited clinical information (e.g., age, age at onset, gender, and family history) (Nalls et al., 2014) or perform deep clinical phenotyping in cohorts not genetically assessed (Lerche et al., 2016). Here, the implementation of the new NeuroChip technology allows the comprehensive testing of all currently known disease genes and risk variants related to the most common neurodegenerative disorders (Blauwendraat et al., 2017). Therefore also potential overlap of pathomechanisms between different neurological diseases may be detected, as recently shown for PD, frontotemporal dementia with parkinsonism (FTDP) and Alzheimer’s disease (AD) (Ferrari et al., 2017). Indeed different forms of parkinsonism with a shared molecular background have been identified, e.g., mutations in the LRRK2 gene were described as causing typical PD, MSA or PSP with histopathological features ranging from synucleinopathies up to tau aggregation (Zimprich et al., 2004).

To define our cohort, we referred to the UK PD brain bank criteria, as the new MDS criteria were not available at the time defining the protocol of our study (Postuma et al., 2015a). As the harmonization and validation of our dataset according to different scales is a major aim of our study, we will perform comparisons of the different diagnostic criteria to delineate potential differences in sensitivity and specificity.

The strategy to include all stages of PD and atypical forms of parkinsonism opens new avenues to investigate the longitudinal course of the respective diseases, and to define predictors of conversion between typical and atypical parkinsonism. However, this also implies the risk of loss to follow-up due to increased morbidity and mortality in advanced disease stages (Fielding et al., 2016). To reduce this risk we included a flexible participation principle either by a flying team allowing patients to participate close to their home environment or, for the most disabled, offering a phone interview with reduced datasets instead of a visit.

We have also demonstrated the feasibility of recruiting PD and atypical parkinsonism in one study. Due to the relatively low prevalence of atypical parkinsonism, considered thus as orphan diseases, there is a lack of population-based comprehensive data for direct comparison to PD (Wenning et al., 2013). As this frequently imposes problems for differential diagnosis, especially in early disease stages, our strategy not only provides different control groups (healthy and diseased) and avoids the drop out of PD patients developing atypical symptoms over time opposed to other ongoing studies (Petrovic et al., 2012). This integrative approach enables us to compare the different forms of parkinsonism, and thus to identify new disease markers (e.g., in the cognitive domain) that could lead to a more accurate differential diagnosis at earlier disease stages.

The deep phenotyping approach implemented in our study by management of big data enables us to adopt a data driven approach, compared to, e.g., other studies investigating prodromal PD that are focusing on theory driven aspects which can imply limitations. Theory-driven research explores in much detail RBD, considered to be a prominent forerunner syndrome marker (Postuma et al., 2015b). Even if the risk of PD is highly increased in individuals with an RBD (30% after 3 years to 66% after 7.5 years), its frequency in PD is not clear. A recent meta-analysis found a pooled prevalence 42.3% of RBD in PD (Zhang et al., 2017). Furthermore, RBD is also found in other synucleinopathies like MSA (Zhang et al., 2017) or DLB (Fereshtehnejad et al., 2017). This discrepancy is leading to a bias in disease characterization, because risk cohorts are representing a specific profile and not reflecting the whole spectrum of parkinsonian syndromes. Here a more data-driven approach will enable us to address new concepts for risk cohorts including the whole spectrum of the disease and avoid biases due to theory-driven approaches.

The multilingual background of participants in our study allows for (i) further investigating the concept of cognitive reserve in neurodegenerative diseases, a concept postulated previously in Luxembourg (Perquin et al., 2013, 2015), and (ii) for the validation of screening tools across languages and provides opportunities for expanding the internationally available tools for clinical research in the area of PD [e.g., validation of the French Munich Dysphagia Test (Hipp et al., 2017)].

As a monocentric study, an advantage over the majority of other nation-wide cohort studies is the low variability and high quality of collected data because of the defined number of raters.

Our program has been designed specifically to allow international collaborations and we successfully implemented shared datasets within REDCap across study centers in Oxford (OPDC) and Tübingen (ABC-PD). In this context, we set up an infrastructure for accepting applications for data sharing, sample sharing and group intellectual sharing that will foster international collaborations.

Among the limitations of our current approach is the lack of a comprehensive imaging and brain banking program that allow for structural and functional image analyses and histopathological confirmation of the diagnosis. This was already partly recognized and the first brain banking program in Luxembourg is currently set up and will allow study participants to donate their brains for research.

The Luxembourg Parkinson’s study will provide a valuable contribution to the international ongoing cohorts and allow cross-validation of emerging results for stratification in PD. Indeed first studies in large cohorts of PD patients allowed to identify “new” subtypes of PD with differing courses of disease progression (von Campenhausen et al., 2005). This was possible due to integration of clinical and biological data and allowed the identification of a more malignant form of PD with more pronounced dopaminergic deficit, increased brain atrophy and an Alzheimer’s disease-like profile of cerebrospinal fluid that was related to faster progression of motor and cognitive deficits. These findings require validation in independent cohorts across different populations worldwide.

Our preliminary results underscore the feasibility of the study considering the efficiency of the actually adapted strategies and estimation of the population composition in the investigated region, and the realization of this cohort study will be efficient and provide comprehensive data on PD in many aspects. Moreover, the well-characterized patients with PD and atypical parkinsonism in our study will open access to new therapies via more focused clinical trials disease subgroups in the future.

## Author Contributions

GH conceived, organized, and executed the research project; executed the statistical analysis; and wrote the first draft, reviewed, and critically revised the manuscript. MV designed, executed, reviewed, and critically evaluated the statistical analysis; and wrote the first draft, reviewed, and critically revised the manuscript. ND and RB conceived the research project, and reviewed and critically revised the manuscript. KR, VS, and PB conceived and executed the research project, and wrote the first draft, reviewed, and critically revised the manuscript. ES and SS wrote the first draft, reviewed, and critically revised the manuscript. SB, LL, AS, BN, and A-MH executed the research project. PK, DR, LP, CP, and LG conceived, organized, and executed the research project, and reviewed and critically revised the manuscript. KM, FB, JK, TG, and MH reviewed and critically revised the manuscript. MG conceived and organized the research project, and reviewed and critically revised the manuscript. RK conceived, organized, and executed the research project; designed, executed, reviewed, and critically evaluated the statistical analysis; and wrote the first draft, reviewed, and critically revised the manuscript.

## Conflict of Interest Statement

RK serves as Editorial Board Member of the European Journal of Clinical Investigation and Journal of Neural Transmission. RK has received research grants from Fonds National de Recherche de Luxembourg (FNR; PEARL [FNR/P13/6682797/Krüger] and NCER-PD [FNR/NCER13/BM/11264123]), the German Research Council (DFG; KR2119/8-1), the Michael J Fox Foundation, the European Union’s Joint Program-Neurodegenerative Diseases (JPND; COURAGE-PD), the European Union’s Horizon2020 research and innovation program (WIDESPREAD; CENTRE-PD; grant agreement no. 692320; CENTRE-PD to RK) and the Federal Ministry for Education and Research (BMBF; Mito-PD 031 A 430 A), as well as speaker’s honoraria and/or travel grants from Abbvie and Medtronic. RK participated as PI or site-PI for industry sponsored clinical trials without receiving honoraria. The remaining authors declare that the research was conducted in the absence of any commercial or financial relationships that could be construed as a potential conflict of interest.

## Footnotes

1 https://www.cdc.gov/epiinfo/index.html

2 https://ada.parkinson.lu

3 www.geopd.net
