# Supplementary material for: The Luxembourg Parkinson’s Study: A Comprehensive Approach for Stratification and Early Diagnosis
Source: Front Aging Neurosci. 2018 Oct 29;10:326. doi: 10.3389/fnagi.2018.00326 (PMC6216083; doi:10.3389/fnagi.2018.00326)
Supplement: Supplementary file 1 [file Table_1.DOCX]

***Supplement: Biosampling***

***Table A: Biospecimen collection and processing***

|  | **Biospecimen Type** | **Primary Container** | **# Specimens per visit** | **Process** | **Derivatives** | **SPREC (specimen or simple derivative)** | **# aliquots x volume** |
| --- | --- | --- | --- | --- | --- | --- | --- |
| Mandatory | Venous blood | 10 ml CAT | 1 | Serum Aliquoting | Serum | SER-CAT-A-B-N-D-A | 12 x 220 µl |
|  |  | 10 ml EDTA | 2 | Blood Processing for Plasma Aliquoting | Plasma EDTA | PL1-PED-A-B-N-D-A | 24 x 220 µl |
|  |  |  |  |  | Whole blood | BDL-PED-A-B-N-D-A | 2 x 300 µl |
|  |  |  |  | Automated DNA Extraction from Non-Fixed Materials | Lysed buffy coat | BFF-PED-A-B-N-D-Z* | 2 x 950 µl |
|  |  | 8 ml CPT | 1 | Isolation and Cryopreservation of Peripheral Blood Mononuclear Cells (PBMCs) | PBCMs | CEL-CPT-A-B-B-A-C | 1 x 1.6 ml |
|  |  |  |  | Blood Processing for Plasma Aliquoting | Plasma Citrate | PL1-CPT-A-B-N-D-A | 12 x 220 µl |
|  |  | 2.5 ml PAXgene | 1 | Automated RNA Extraction from Non-fixed Materials | RNA | BLD-PAX-C,E,G,I,K | 1 x 77 µl |
|  | Mid-stream urine | 50 ml sample, no additive | 1 | Urine centrifugation and aliquoting | Urine pellet | PEN-PPS-B-J-N-D-A | 1 x 1250 µl |
|  |  |  |  |  | Urine supernatant | URN-PPS-B-J-N-C-J | 12 x 3.8 ml |
|  | Saliva | OMNIgene saliva system | 1 | Nucleic Acid Extraction from Fluids | DNA | SAL-ORG-C,E,G,I,K | 1 x 120 µl |
| Optional | Stool | OMNIgene.GUT stool tube | 1 | Stool aliquoting | Stool | STL-ZZZ**-M-N-N-B-Y | 2 x 1.8 ml |
|  |  |  |  | Automated DNA extraction from non-fixed materials | DNA |  | 1 x 0.5 ml |
|  | Cerebrospinal fluid (CSF) | 11 ml NUNC conical tubes (First specimen of 2 x 2 ml – Clinical CSF Assessment) | 2 | Clinical assessment | N/A | N/A | N/A |
|  |  | 3.5 ml BD SST II Advance Tube – Clinical Assessment | 1 | Clinical assessment | N/A | N/A | N/A |
|  |  | 10 ml Sarstedt tube (Second specimen of 8 ml – Biobanking) | 1 | CSF Processing | CSF pellet | PEN-PPS-A-B-N-B-A | 1 x 100 µl |
|  |  |  |  |  | CSF supernatant | CSF-PPS-A-B-N-B-A | 20 x 500 µl |
|  | Skin biopsy | 2 ml cryovial filled with 1.8ml sterile Transport Media(DMEM/Glutamax/PenStrep) | 1 | Fibroblast culture | Passage 3 Fibroblasts | TIS-BSL-N-F-ZZZ***-N-Z*** | 10 x 1-2 x 10^6^ fibroblasts |

* no long term storage

******original: OMNIgeen GUT tube (DNA genotec).

*** processing for fibroblast culture

***Table B: Sample Quality Control and characterization***

|  | Type of specimen or derivative | Quality Control and characterization method |
| --- | --- | --- |
| Specimen | Whole blood | Complete Blood Count with the ABX Micros CRP measurement |
|  | CSF | Cell count, IgG, IgA, IgM, albumin, lactate |
|  | Urine | Urine analysis parameters : Leukocytes, pH, protein, glucose, ketone, urobilinogen, billirubin,density, nitrites, specific gravity, blood, Hemoglobin, |
| Derivative | Blood DNA | Quantification by spectrophotometry, purity by spectrophotometry, spectrofluorometry* |
|  | Blood RNA | Quantification by spectrophotometry, purity by spectrophotometry, RIN* |
|  | Blood PBCM | Enumeration, viability |
|  | Stool DNA | Quantification by spectrophotometry, purity by spectrophotometry, spectrofluorometry*, 16S rRNA gene sequencing |
|  | Saliva DNA | Quantification by spectrophotometry, purity by spectrophotometry, spectrofluorometry* |
|  | EDTA plasma | Hemoglobin*, LacaScore*, Cytokine* |
|  | CPT Plasma | Hemoglobin*, Cytokine* |
|  | Serum | Hemoglobin*, CD40L*, Cytokine* |
|  | Urine supernatant | cysC, creatinine* |
|  | Fibroblast | Enumeration, viability |

*assays performed on demand
